# Mentor and mentee experiences of the NMC-CBME mentorship programme in India: a two-centre qualitative study

**DOI:** 10.1186/s12909-026-10132-x

**Published:** 2026-08-11

**Authors:** Amrita Sarkar, Jarina Begum, Debjit Roy, Swati Shikha, Syed Irfan Ali

**Affiliations:** 1 Department of Community Medicine, Tomo Riba Institute of Health and Medical Sciences (TRIHMS), Naharlagun, Arunachal Pradesh India; 2 Department of Community Medicine, Manipal Tata Medical College, Manipal Academy of Higher Education, Manipal, Karnataka India; 3 Department of Psychiatry, IQ City Medical College, Durgapur, West Bengal India

**Keywords:** Mentorship, Medical education, CBME, NMC, Qualitative research, MBBS, India, Focus group discussion, In-depth interview, Thematic analysis

## Abstract

**Background:**

India’s National Medical Commission mandated a formal mentorship programme within competency-based medical education for all MBBS students. However, evidence regarding how mentors and mentees experience this programme remains limited. This study explored the perceptions and experiences of both groups across two geographically and demographically distinct tertiary-care institutions.

**Methods:**

A two-centre parallel qualitative study was conducted at Site A (Jharkhand) and Site B (Arunachal Pradesh). Mentee perspectives were collected through eight focus group discussions (four/site) with MBBS students. Mentor perspectives were explored through in-depth interviews (15 at Site A & 24 at Site B). Focus Group Discussion (FGD) data were analysed using inductive thematic analysis (Braun and Clarke), while In Depth Interviews (IDI) data were analysed using framework analysis. Site-level analyses were conducted independently and integrated through cross-site thematic comparison. Reporting followed COREQ guidelines.

**Results:**

39 mentors and 56 mentees participated. Nine thematic domains emerged. Eight were convergent across both sites: structural and implementation inadequacies; scheduling deficiencies and boundary violations; differential academic support quality; psychosocial support potential constrained by structural barriers; mentor unpreparedness and quality variability; hierarchical communication barriers; phase-specific mentorship needs; and an accountability vacuum with absent feedback mechanisms. One theme (cultural and contextual specificity) was unique to Site B. Across all data sources, participants endorsed mentorship in principle but described implementation as structurally inadequate. Informal communication channels and social interactions, organically adopted at Site B, appeared to mitigate some gaps that participants at Site A identified as desired improvements.

**Conclusions:**

This study concludes that the convergence of findings across two sites suggests that these implementation challenges may reflect broader features of the programme; however, this interpretation should be considered an analytical inference requiring confirmation in broader settings. Strengthening mentorship requires protected time, structured mentor training, explicit confidentiality safeguards, separation of mentoring from summative evaluation, and institutional recognition. Findings further indicate that context-sensitive adaptation is essential, particularly for minority and tribal student populations.

**Supplementary Information:**

The online version contains supplementary material available at https://doi.org/10.1186/s12909-026-10132-x.

## Introduction

Medical education in India underwent a landmark structural transformation with the introduction of competency-based medical education (CBME) by the National Medical Commission (NMC). Within this framework, a formal mentorship programme was mandated for all undergraduate medical students, with each MBBS student assigned a faculty mentor responsible for providing academic guidance, psychosocial support, and longitudinal developmental oversight throughout the five-and-a-half-year programme [1]. The intent was to embed a structured, individualised support relationship into an educational environment that has historically emphasised didactic instruction over mentored professional formation.

Mentorship in medical education has a well-established evidence base. Effective mentoring relationships are associated with improved academic performance, enhanced professional identity formation, better mental health outcomes, and greater career satisfaction among medical students [2, 3]. Inadequate mentorship, characterised by infrequent contact, role ambiguity, hierarchical power differentials, and the absence of institutional support, produces few measurable benefits and may generate additional burden for both mentors and students [4, 5]. 

Mentorship is widely recognised as a cornerstone of health professions education, fostering professional development, career satisfaction, research productivity, and the transition from theory to practice. Effective mentoring is grounded in mutual trust, structured guidance, reflective practice, and institutional support; however, its implementation is often constrained by time pressures, mentor workload, and limited organisational recognition, highlighting the need for formal mentor training and sustained institutional commitment [6, 7]. 

Mentorship is a dynamic, reciprocal relationship that provides both career and psychosocial support, fostering holistic professional and personal development. Unlike advising or coaching, it is a learned skill that evolves through sustained engagement between mentor and mentee. Studies have found that faculty recognised mentorship as valuable for professional development; however, mentoring was largely informal and constrained by limited training, heavy workloads, and the absence of structured institutional policies and recognition for mentors [8, 9]. 

Evidence from Indian medical colleges suggests that structured mentorship programmes positively influence students’ academic performance, professional identity, self-discipline, research motivation, and personal development. However, their effectiveness is often constrained by limited dedicated time, infrequent mentor–mentee interactions, and competing academic commitments, highlighting the need for protected mentoring time, smaller mentor–mentee groups, regular programme evaluation, and the judicious use of digital communication platforms to strengthen engagement [10]. 

The NMC mandate, while progressive in intent, has proceeded without a parallel infrastructure of implementation guidance, monitoring frameworks, or faculty development. Studies from early adopter institutions suggest that programme delivery varies substantially depending on institutional culture, administrative commitment, and individual faculty motivation [11, 12]. What has not been adequately characterised is how these structural factors are experienced on the ground: specifically, how both mentors and students narrate their participation in the programme across institutions operating under materially different conditions.

This study was designed to address that gap. Using a qualitative two-centre design, we examined the perceptions and experiences of both mentors and students in two tertiary-care teaching institutions: one in Jharkhand (Site A) and one in Arunachal Pradesh (Site B). The two institutions were purposively selected because they differed substantially in programme maturity, geography, institutional resources, and student demographics, thereby providing an opportunity to explore whether implementation challenges converged across contrasting educational contexts.

The research questions guiding the study were: (1) How do mentors and students experience the NMC-CBME mentorship programme in their institutions? (2) What structural, relational, and contextual factors shape these experiences? (3) How do these factors converge or diverge across two geographically distinct sites?

## Methods

### Study design

This was a two-centre qualitative study using in-depth interviews (IDIs) with mentors and Focus Group Discussion (FGDs) with mentees, conducted in parallel at two geographically distinct sites and subsequently integrated through cross-site thematic comparison. Individual in-depth interviews were conducted with mentors to facilitate confidential discussion of institutional responsibilities, personal experiences, and implementation challenges that participants might have been reluctant to express in a group setting. Focus group discussions were used with mentees because shared interaction encouraged participants to reflect on common experiences, compare perspectives, and build upon each other’s observations, thereby generating richer insights into the functioning of the mentorship programme. Each site collected and analysed data independently before integration, which reduces the risk of one site’s framing unduly influencing interpretation at the other. Mentee FGD transcripts at both sites were analysed using an inductive thematic analysis informed by Braun and Clarke’s six-phase approach [13]. Mentor IDI transcripts at both sites were analysed using framework analysis supported by inductive coding. Framework matrices were developed iteratively and subsequently compared across sites. The two sites differ substantially in geography, student demographics, programme maturity, and institutional resources. This non-equivalence is treated as analytically informative rather than as a methodological limitation: convergence of challenge profiles across non-equivalent sites carries stronger inferential weight than convergence across similar ones, while divergent findings illuminate the role of local context.

Mentor and mentee datasets were analysed separately as they represented distinct stakeholder groups with different roles, experiences, and expectations within the mentorship programme. Separate analyses enabled preservation of role-specific meanings before undertaking cross-site and cross-source integration to identify convergent and divergent patterns across the complete dataset.

All interpretations derived from the cross-site comparison are framed as analytical inferences from two sites and not as generalisable claims about the NMC programme as a whole. Reporting follows COREQ [14]. The same has been detailed in the supplementary file.

### Setting

Site A was the Department of Community Medicine at a tertiary-care teaching hospital in Jamshedpur, Jharkhand. Site B was the Department of Community Medicine at a tertiary-care teaching hospital in Naharlagun, Arunachal Pradesh. Both institutions implement the NMC-CBME mentorship programme as mandated. Site A represents a programme with greater operational maturity. Site B has a distinct demographic context: a student body drawn substantially from geographically remote tribal communities, many of whom are first-generation college-goers.

### Participants

Eligible mentors were MBBS faculty members identified from institutional mentorship records who had actively served as assigned mentors under the NMC-CBME mentorship programme and had sufficient experience of the mentoring process to reflect on programme implementation, and eligible mentees were MBBS students across Phases I to III identified from the official mentor–mentee allocation lists who had participated in the mentorship programme for an adequate duration to describe their experiences.

Faculty without mentoring responsibilities and administrative personnel not directly engaged in mentoring were excluded. Newly assigned mentees with minimal mentor interaction, interns, and students who had not participated in the mentorship programme were not included.

Participants were recruited purposively to ensure diversity in gender, academic phase, batch, and mentoring experience. Phase I comprises the first professional year, Phase II includes the second professional year, and Phase III encompasses the subsequent clinical training years (Part I and Part II) leading to completion of the MBBS programme.

Participants were approached by trained research team members of both genders with a postgraduate degree who work at the institute as full-time regular faculty. Wherever feasible, recruitment and interviews/focus group discussions were conducted by investigators in the presence of faculty who were not directly responsible for participants’ academic assessment or routine supervision.

Purposive sampling followed a maximum variation approach at both sites to include participants face-to-face with diverse characteristics, including gender, academic phase, batch, and mentoring experiences among students, and variation in academic rank, department, and mentoring experience among faculty.

Fifteen mentor IDIs were conducted at Site A (coded A-IDI-M1 to A-IDI-M15); 24 mentor IDIs were conducted at Site B (coded B-IDI-M1 to B-IDI-M24). Mentee FGD participation comprised 28 students across four FGDs at Site A (A-FGD1 to A-FGD4) and 28 students across four FGDs at Site B (B-FGD1 to B-FGD4).

### Participant coding

A unified site-specific coding system is used throughout this manuscript. FGD participants are identified by site prefix, FGD number, and participant number: Site A participants as A-FGD [1–4]-P[n]; Site B participants as B-FGD [1–4]-P[n]. IDI participants are identified as A-IDI-M [1–15] (Site A) and B-IDI-M [1–24] (Site B). All codes are defined here and used consistently in quotations and tables throughout.

### Data collection

IDIs & FGDs were conducted at mutually convenient times in consultation with participants, outside scheduled teaching sessions or clinical duties whenever possible.

IDIs, FGDs were conducted by trained members of the research team using a semi-structured topic guide & piloted before use; a research assistant documented group dynamics and emotional tenor concurrently. The semi-structured IDIs & FGDs were developed by the research team following a review of the literature on mentorship in competency-based medical education and were aligned with the study objectives. The FGD guide explored mentees’ experiences across key domains, including mentor–mentee matching, relationship dynamics, learning and skill development, communication, perceived barriers, programme improvement, mentor training, and personal growth. The IDI guide was designed to explore mentors’ perspectives on their needs, strengths, challenges, recommendations for programme improvement, and personal reflections on the mentoring role. Prior to formal data collection, both guides were reviewed by the investigators for content relevance, clarity, logical sequencing, and comprehensiveness, and were piloted with participants who met the eligibility criteria but were not included in the final analysis. Feedback from the pilot resulted in minor refinements to the wording, ordering of questions, and incorporation of additional probes to facilitate richer discussion while preserving the overall structure of the guides. The same core domains and principal questions were used across both study sites to ensure comparability; however, interviewers were permitted to use context-specific probing questions to explore emerging issues relevant to local programme implementation without altering the substantive content of the guides. The final versions of both guides are provided as Supplementary File 1.

All interviews and FGDs were audio-recorded with participant consent and transcribed verbatim. FGDs at Site B lasted between 85 and 100 min; FGDs at Site A lasted between 75 and 95 min. The IDI guide was adapted at each site to reflect local programme context while maintaining comparability of core domains across sites.

Interviews and focus group discussions were conducted primarily in English, with participants occasionally using Hindi or local language expressions to facilitate natural communication. Audio recordings were transcribed verbatim, and where translation into English was required, bilingual members of the research team translated and cross-checked the transcripts against the original audio recordings to ensure conceptual accuracy and preserve the intended meaning. Any discrepancies identified during translation or transcription were resolved through discussion and consensus among the investigators before analysis.

### Analysis

Data collection and analysis proceeded concurrently. Recruitment continued until thematic saturation was achieved independently at each site, defined as the point at which additional interviews or focus group discussions generated no substantively new codes or themes. All transcripts were checked against the original audio recordings by members of the research team to ensure transcription accuracy before analysis commenced.

FGD transcripts at both sites were analysed using inductive thematic analysis following Braun and Clarke [13]. IDI transcripts at both sites were analysed using framework analysis combined with inductive thematic analysis.

Thematic saturation was assessed independently at each site using the criterion of no substantively new themes or sub-themes emerging from additional transcripts: at Site A, saturation was confirmed after A-FGD4 and the 15th IDI; at Site B, saturation was confirmed after B-FGD4 and the 24th IDI. At each site, two analysts coded transcripts independently before comparing and reconciling codes through structured discussion; disagreements were resolved by consensus and, where persistent, escalated to a third team member. An audit trail documenting coding decisions, theme evolution, and integration steps was maintained throughout and informed the final thematic structure. The analytical framework was iteratively refined across three rounds of team review prior to finalisation. No formal member reflection or participant validation exercise was conducted; this is acknowledged as a limitation. A detailed coding tree illustrating the progression from initial codes to themes has been included in Supplementary File 2.

All faculty and student participants at both sites agreed to take part when approached; no approached participant declined participation. Although all approached participants consented to participate after receiving the participant information sheet, with detailed information regarding voluntariness and confidentiality, the hierarchical nature of medical education may have influenced participation decisions or contributed to social desirability in participant responses despite the safeguards employed.

### Reflexivity

IDIs and FGDs were conducted by trained members of the research team at their respective institutions, all of whom had backgrounds in Community Medicine, Public Health, Medical Education, and prior experience in qualitative research. Although the investigators served as faculty members and mentors within the institutional mentorship programme, none were assigned mentors, academic assessors, or supervisors of the student participants included in the FGDs or the faculty members participating in the IDIs. The research team acknowledged that their professional interest in mentorship and medical education could influence data collection and interpretation. To enhance reflexivity and minimise potential bias, facilitators employed reflective probing techniques to encourage critical and balanced perspectives, remained attentive to potential social desirability during interviews and discussions, and avoided leading questions. A separate research assistant documented group dynamics during FGDs to facilitate contextual interpretation. Independent coding by two researchers, consensus discussions, maintenance of an audit trail, iterative refinement of themes, and independent analysis at each site before cross-site integration were undertaken to challenge assumptions and ensure that the findings remained grounded in participants’ accounts rather than researchers’ preconceptions.

### Ethical approval

Ethical approvals for Site A and Site B were obtained from the Institutional Ethics Committee (reference: MTMC/IEC/2024/72 and TRIHMS/IEC/05/2023 Ref. file IEC/2023/075). Both approvals were obtained for the study protocol titled “Perceptions and Experiences of Mentors and Mentees in an MBBS Mentorship Programme: A Mixed Method Study”; the present manuscript reports the qualitative component of that approved protocol. All participants provided written informed consent prior to data collection. Participants were explicitly informed that participation was voluntary and that declining or withdrawing would have no effect on their academic progression, employment, assessment, or mentor–mentee relationship. Participant confidentiality was maintained throughout; all quotations are identified by FGD session code, participant code, or site only.

## Results

### Participant overview

A total of 39 mentors participated in IDIs across both sites: 15 at Site A (Jharkhand) and 24 at Site B (Arunachal Pradesh). Mentee perspectives were gathered through FGDs: 28 students participated across four FGDs at Site A (A-FGD1 to A-FGD4, Phases I to III), and 28 students participated across four FGDs at Site B (B-FGD1 to B-FGD4, Phases I to III). Table 1 summarises participant numbers, FGD composition, and analytical methods across both sites.

**Table 1:** Participant characteristics and FGD composition across both sites

Parameter	Site A: Jharkhand	Site B: Arunachal Pradesh	Method	Total
Mentors (IDI)	15 (A-IDI-M1 to A-IDI-M15)	24 (B-IDI-M1 to B-IDI-M24)	In-depth interviews	39
Mentees (FGD)	28 students; 4 FGDs (A-FGD1 to A-FGD4); Phases I to III (male: 14, female: 14)	28 students; 4 FGDs (B-FGD1 to B-FGD4); Phases I to III (male: 15, female: 13)	Focus group discussions	56
Analytical approach	Inductive thematic analysis (FGD); framework analysis combined with inductive thematic analysis (IDI)	Inductive thematic analysis (FGD); framework analysis combined with inductive thematic analysis (IDI)		
Thematic saturation	Confirmed after A-FGD4 and the 15th IDI	Confirmed after B-FGD4 and the 24th IDI		

FGD Composition: Site A (Jharkhand)				
FGD	*n*	MBBS Phase and Key Features		
A-FGD1	7	Phase I; initial adjustment to MBBS curriculum; orientation to mentorship programme; first-year transition experiences		
A-FGD2	6	Phase II; academic stressors and scheduling conflicts prominent; reduced meeting frequency noted		
A-FGD3	7	Phase III (Part 1); continuity disruptions and specialty-phase mismatch raised; mentor quality variability foregrounded		
A-FGD4	8	Phase III (Part 2), clinical years; most senior group; retrospective and career-guidance perspectives; thematic saturation confirmed		
**Total**	**28**	**Phases I to III; male: 14**,** female: 14**		

FGD Composition: Site B (Arunachal Pradesh)				
FGD	*n*	MBBS Phase and Key Features		
B-FGD1	7	Phase I, Batch 3; first batch to receive formal programme orientation; structured first impressions captured		
B-FGD2	6	Phase I, Batches 1 and 2; no formal orientation received; most acute accounts of structural dysfunction		
B-FGD3	8	Phase II; continuity disruptions prominent; Phase II academic stressors raised		
B-FGD4	7	Phase III, clinical years; most senior group; longitudinal and retrospective perspective; thematic saturation confirmed		
Total	28	Phases I to III; male: 15, female: 13		

*IDI *in-depth interview, *FGD  *focus group discussion. Thematic saturation was assessed independently at each site using standard criteria. C = convergent; A only = Jharkhand-specific. All FGDs were audio-recorded with participant consent. A research assistant documented group dynamics concurrently. The topic guide was semi-structured and piloted before use. Site B, B-FGD1 (March 2026) represents the first batch to receive formal programme orientation, in contrast to B-FGD2 to B-FGD4 (November 2024 to April 2025), where no formal orientation had been provided; this natural variation provided analytically significant comparative data within Site B

### Thematic findings: mentee perspectives (focus group discussions)

**Fig. 1:**
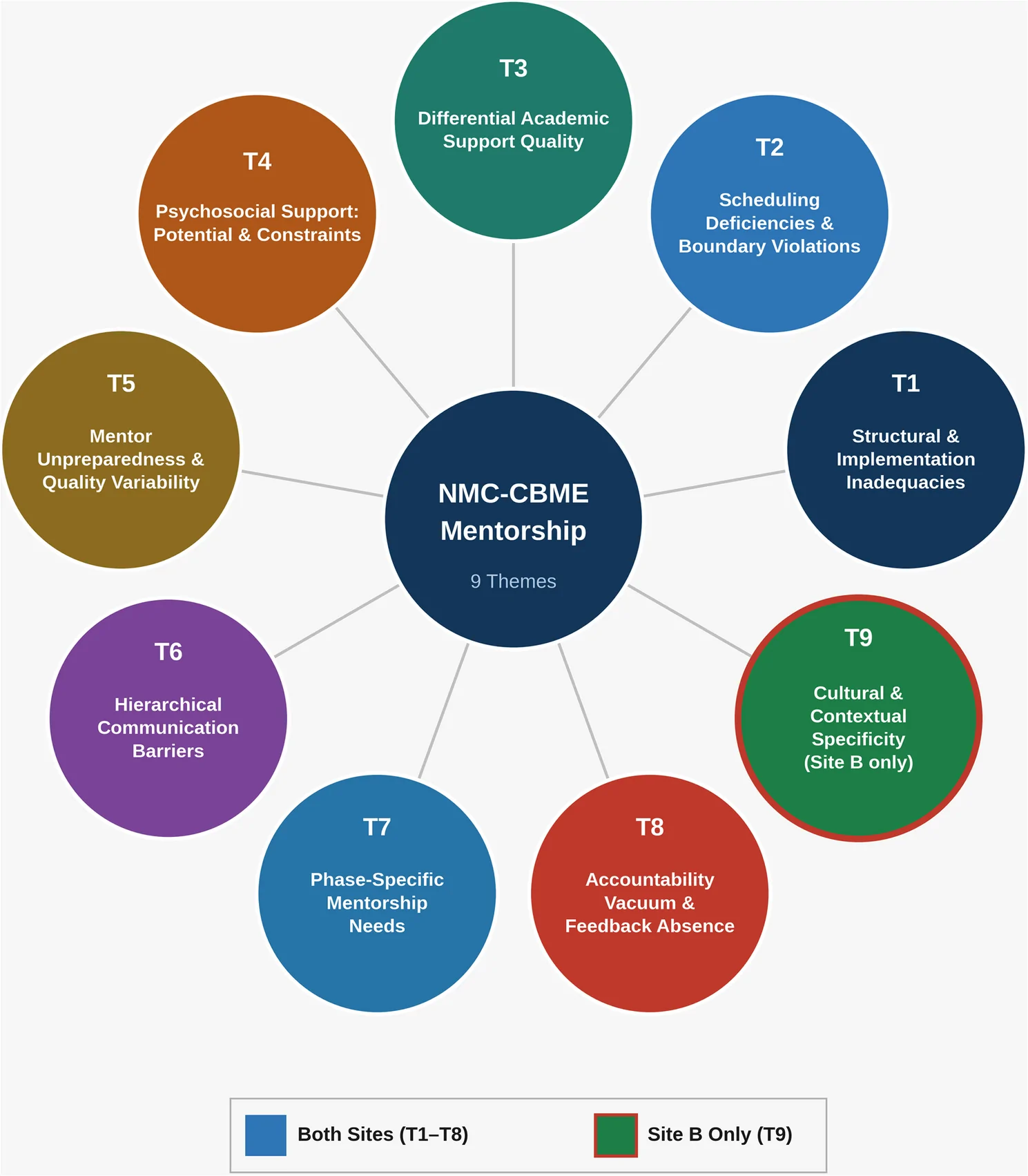
Thematic framework: nine domains emerging from integrated cross-site analysis. Themes T1 to T8 were convergent across both sites; T9 (cultural and contextual specificity) was unique to Site B (Arunachal Pradesh)

Inductive thematic analysis of FGD transcripts, following Braun and Clarke, yielded nine overarching themes across the two sites. Eight were convergent across both sites; one (Theme 9: cultural and contextual specificity) was unique to Site B. Themes T1 to T8 are presented below; their distribution across both sites is illustrated in (Fig. 1).

#### Theme 1: structural and implementation inadequacies

At both sites, students identified a systematic gap between the stated programme architecture and its operational delivery. At Site B, students from early batches described a condition of programmatic invisibility: the programme had been assigned without explanation of its existence, scope, or expectations. At Site A, structural gaps were expressed primarily through scheduling irregularity and inadequate attendance enforcement, with perceived programme seriousness linked directly to whether attendance was formally required.

*We received a notice at some point with a faculty name and phone number. We went to introduce ourselves over the phone. We spoke for about ten minutes. That was the beginning and also*,* for several months*,* the end.* (B-FGD2-P4, Site B)

*Since there is no mandatory attendance*,* everybody takes it lightly.* (A-FGD2-P3, Site A)

The contrast between batches at Site B was analytically significant: Batch 3 students who received a structured orientation experienced the programme as a legitimate institutional commitment, while prior batches experienced it as a bureaucratic formality. Orientation thus functioned not merely as information transfer but as institutional legitimation.

#### Theme 2: scheduling deficiencies and boundary violations

Meeting frequencies far below the stated fortnightly standard were reported at both sites. The typical range was three to five meetings per academic year. Students at both sites attributed infrequency to faculty workload rather than personal disinterest, framing the problem as structural rather than interpersonal.

*It sounds acceptable*,* but real-world implementation is different. Specific time is essential. If mentoring meetings always compete with other duties*,* then neither the faculty nor the students will be able to find time.* (B-FGD3-P3, Site B)

A finding unique to Site B was the scheduling of meetings on Sundays or after 6 PM. Students identified Sunday as their only unscheduled day, used for rest, family contact, and recovery. The power differential made refusal feel risky, yet compliance meant sacrificing their only genuine rest period, representing a boundary violation with both relational and welfare dimensions.

*Sunday is our only full rest day. If a mentor suggests meeting on a Sunday it puts you in a difficult position because you feel you should say yes*,* but it is genuinely the only time in the week when you can rest*,* call your family*,* do your laundry.* (B-FGD1-P6, Site B)

Across all four FGDs at Site B, students independently proposed the same structural solution: a designated meeting slot within the institutional timetable, within working hours, non-optional for both parties. At Site A, students proposed that although the meeting timings are fixed, the meeting process should be standardised and held in a common venue, such as a lecture theatre or demo rooms.

#### Theme 3: differential academic support quality

Student experiences of academic support were widely distributed at both sites, ranging from transformative to entirely absent. At Site B, the distinguishing variable was mentor proactivity: mentors who asked specific questions about specific assessments generated accountability, while those who waited for students to raise issues received a universal response of being fine. Meta-learning guidance was particularly valued, especially when grounded in the mentor’s own curriculum experience.

*My mentor identified that I am a visual learner. I did not even know this about myself. She recommended mind-mapping. It transformed my preparation.* (B-FGD3-P3, Site B)

*She advised me to talk to teachers about how to study the subject.* (A-FGD1-P2, Site A)

Faculty disclosure of their own academic difficulties was identified at Site B as among the most impactful relational moments, normalising the experience of struggle and challenging the implicit assumption that successful faculty members have always performed well. Career guidance and professional networking were identified as important by Phase III students at Site B, who recognised that faculty connections were something junior students could not independently access.

*My mentor connected me with a senior resident in a department I am interested in. That networking function is something a student cannot do on their own*,* especially as a junior student with no established relationships among the faculty.* (B-FGD4-P7, Site B)

Both sites independently identified specialty-phase mismatch as a structural limitation: when the mentor’s specialty was unrelated to the student’s current academic phase, the academic utility of the assigned relationship was constrained, regardless of the mentor’s willingness to engage.

#### Theme 4: psychosocial support, potential and structural constraints

Both sites recognised psychosocial support as a legitimate programme function, yet structural barriers consistently prevented this potential from being realised. At Site B, the most analytically significant barrier was the concurrent evaluator-mentor role: students reported that their mentor’s concurrent role as examiner and referee led to conscious self-censorship, even where they expressed positive regard for their mentor. The extent to which this role overlap affects disclosure is likely to vary by institutional culture and individual circumstance, and should be understood as a context-specific structural concern rather than a universal feature of mentorship programmes.

*My mentor is also an examiner for our internal assessments. That fact alone makes me careful about how much vulnerability I show. I like her*,* I trust her to some extent*,* but I cannot be fully open because of that.* (B-FGD3-P2, Site B)

At Site A, inhibition was framed primarily in terms of age hierarchy and administrative seniority. An additional finding specific to Site A was parental engagement by the mentor: direct communication with students’ families was documented as a valued programme feature, representing a distinctive contextual adaptation.

*My mentor talked to my father and counselled both of us.* (A-FGD3-P1, Site A)

Trust was understood at both sites as a developmental outcome requiring consistent engagement over time. In a programme where most dyads met three to five times per year, the trust threshold required for personal disclosure was rarely reached. Confidentiality ambiguity was an additional deterrent at Site B, with students uncertain about what disclosures could be shared with other faculty or institutional leadership.

*We do not interact with them frequently. How can you open up to someone who does not even know much about you and you do not know much about them either?* (B-FGD2-P3, Site B)

#### Theme 5: mentor unpreparedness and quality variability

Wide variation in mentoring quality was identified at both sites and attributed primarily to individual personality and intrinsic motivation rather than systemic preparation. At Site B, unanswered messages constituted some of the most emotionally significant negative experiences, with students describing disengagement in terms of resigned accommodation rather than anger.

*The first message he sent sounded warm*,* the second had quite a delay in reply*,* and by the third message he did not reply at all. After that I stopped messaging. I felt like he did not want to be disturbed.* (B-FGD2-P2, Site B)

*My mentor was mostly absent or had very little time for interaction.* (A-FGD2-P5, Site A)

Both sites independently identified that mentors did not know what to ask in meetings, producing sessions that defaulted to general social exchange. Students attributed this to an absence of training rather than personal disinterest. Both sites independently noted that programme outcomes varied substantially according to individual mentor initiative, with effective mentoring consistently attributed to personal motivation rather than structural preparation.

*Good mentors do it out of personal commitment*,* which is admirable*,* but not all do the same. It makes the other mentees like me feel unfortunate.* (B-FGD1-P5, Site B)

#### Theme 6: hierarchical communication barriers

Senior-junior hierarchy was identified as a communication inhibitor at both sites. At Site B, the evaluator-role barrier was articulated as a structurally distinct and additional obstacle. At Site A, age and departmental seniority were identified as the primary inhibitors.

*Even now*,* in second year*,* I feel nervous before meetings. There is definitely a power imbalance.* (B-FGD3-P4, Site B)

*Gender is not a barrier*,* but age is. If the mentor is head of department*,* there will be hesitation.* (A-FGD3-P6, Site A)

#### Theme 7: phase-specific mentorship needs

Both sites identified the first year, particularly the initial months, as the period of highest mentorship need. Site A articulated an explicit three-phase model: high need in the first year, reduced perceived need in the middle years, and a renewed career-guidance need in the final year oriented toward postgraduate planning. At Site B, phase-specific variation was present but distributed across other themes rather than consolidated into a distinct sub-framework.

*The first three months are important.* (A-FGD1-P4, Site A)

*In fourth year*,* perhaps we can ask for postgraduate guidance.* (A-FGD4-P2, Site A)

#### Theme 8: accountability vacuum and feedback absence

No mechanism for structured student feedback existed at either site, and no programme outcomes were systematically tracked. At Site B, this constituted a fully developed theme across three codes: absence of a student feedback mechanism, non-measurement of programme outcomes, and institutional relegation of the programme to a compliance exercise. At Site A, the concern was expressed through recommendations for standardisation rather than as an articulated governance critique.

*If only the number of meetings is tracked*,* not what was discussed or whether the mentee benefited.* (B-FGD4-P4, Site B)

#### Theme 9: Cultural and Contextual Specificity (Site B Only)

A theme unique to Site B captured ways in which the specific institutional context shaped the mentoring relationship in ways not fully anticipated by the NMC’s generic programme design. Students across three FGDs clarified that shared tribal identity was not in itself the relevant factor; what was valued was contextual literacy, defined as an understanding of the social and material conditions of students from Arunachal Pradesh.

*Tribal identity does not predict mentor quality. I have never thought: I wish my mentor was from my tribe. I have thought: I wish my mentor would respond to my messages.* (B-FGD2-P6, Site B)

Cultural norms specific to this student population, including the coding of personal disclosure to seniors as weakness or loss of face, further suppressed help-seeking. Students proposed that programme orientation explicitly normalise help-seeking within the mandated support context.

*In this context we do not easily discuss mental health or personal problems with seniors. There is a hierarchy. The programme should address this barrier*,* perhaps through workshops on how to have open conversations.* (B-FGD4-P5, Site B)

One participant described a situation where a mentor defaulted to Hindi idioms the student did not fully understand during an emotionally significant conversation, resulting in a sense that the support had not fully landed. This represents a correctable communicative barrier requiring cultural orientation for mentors assigned to students from linguistic minority backgrounds.

### Thematic findings: mentor perspectives (in-depth interviews)

Analysis of mentor IDI transcripts yielded findings organised across five domains: mentor needs, programme strengths, challenges, recommendations, and personal reflections. At Site A, IDIs were analysed using framework analysis combined with inductive thematic analysis; at Site B, open inductive coding was applied. Table 2 presents the domain-level cross-site comparison.

**Table 2:** Thematic domain overview: mentor IDI findings across both sites

Domain	Site A (Jharkhand)	Site B (Arunachal Pradesh)
Mentor needs	Structured orientation (role and responsibilities); faculty development programme (communication, active listening, conflict resolution); institutional support (protected time, appraisal recognition, logistical infrastructure, referral pathways)	Clarity of expectations (B-IDI-M1); training, time, and support required (B-IDI-M2); unclear scope and absence of time allocation as primary constraints
Programme strengths	Interpersonal and communication skills; professional role modelling; holistic student support (personality development, emotional reassurance, ethical conduct, confidence building)	Informal bonding through social outings effective (B-IDI-M7); WhatsApp as functional primary communication channel (B-IDI-M3, B-IDI-M10, B-IDI-M6)
Challenges	Time constraints and workload; variable student engagement; managing sensitive or complex student issues; lack of standardisation	Workload burden (B-IDI-M4, B-IDI-M3); student disengagement ( *“begging”* metaphor: B-IDI-M1, B-IDI-M4); co-curricular scheduling conflicts (B-IDI-M2, B-IDI-M5); role ambiguity (B-IDI-M5, B-IDI-M9); programme artificiality (B-IDI-M9, B-IDI-M10); financial burden on faculty (B-IDI-M8, B-IDI-M12)
Recommendations	Structured monitoring framework (standard operating procedure, logbook, defined schedules, periodic review); formal training; recognition and incentivisation (appraisal credit, certification); digital and group communication platforms	Better programme design and mentor-mentee matching (B-IDI-M10); institutional administrative backing with adequate time, structure, and resources (B-IDI-M13)
Personal reflections	Professional fulfilment; improved communication skills, empathy, and patience; better understanding of student environment; development of leadership identity	Benefits conditional on programme quality (B-IDI-M11, B-IDI-M14): *“only when the process is genuine and supported”*; confidence and communication gains when genuinely engaged (B-IDI-M14)

Site A: *n* = 15 mentors (A-IDI-M1 to A-IDI-M15); Site B: *n* = 24 mentors (B-IDI-M1 to B-IDI-M24). Site A findings are from framework analysis combined with inductive thematic analysis of 15 IDIs. Site B findings are from open inductive coding of 24 IDI transcripts. Participant codes in parentheses refer to the unified coding system defined in Methods

#### Mentor needs

Site A mentors articulated three structured sub-domains of need: a formal orientation covering role expectations and scope; a faculty development programme addressing communication, active listening, conflict resolution, and sensitisation to generational differences; and institutional support comprising protected time, recognition in appraisal systems, logistical infrastructure, and access to referral pathways for students in psychological distress. At Site B, the same needs emerged in less structured form, with mentors citing unclear expectations, lack of training, and the absence of any time allocation as primary constraints.

*Mentoring is valuable in principle*,* but it only works when there is clarity. Right now*,* the expectations are not very clear*,* and the meetings are not regular. Without that*,* it becomes difficult to say the programme is really functioning as intended.* (B-IDI-M1, Site B)

Across both institutions, mentors consistently perceived that effective mentoring depends less on individual goodwill than on organisational preparedness. Although mentors expressed strong commitment to the mentoring role, they viewed clarity of expectations, faculty development, and institutional support as prerequisites rather than optional enhancements. This pattern suggests that deficiencies in programme implementation are primarily structural rather than attributable to inadequate mentor motivation, reinforcing the importance of institutional investment in sustaining mentoring relationships.

#### Programme strengths

Site A mentors described programme strengths in terms of interpersonal and communication skills, professional experience and role modelling, and holistic student support encompassing personality development, emotional reassurance, ethical conduct, and confidence building. At Site B, the functional strengths identified were primarily informal and adaptive: WhatsApp-based communication and social outings in relaxed settings were independently identified by multiple participants as more effective than formal sessions.

*When we take them out for lunch or dinner*,* or even sometimes for a movie*,* the bonding improves a lot. In a relaxed setting*,* they open up much more. Then the relationship feels real.* (B-IDI-M7, Site B)

*This programme*,* implemented well*,* could be one of the most important things about an MBBS education. It is the one relationship in the institution that is designed entirely around your individual development. Do not waste that.* (B-IDI-M3, Site B)

*WhatsApp works better than scheduling formal meetings every time. We can check on them*,* share guidance*,* and keep communication alive without making it too rigid.* (B-IDI-M10, Site B)

Despite differences in programme maturity between the two institutions, mentors consistently valued mentorship as a relationship-centred educational process extending beyond academic supervision. Notably, Site B mentors developed informal communication strategies such as WhatsApp interactions and social outings as adaptive responses to organisational constraints. Rather than representing isolated innovations, these practices illustrate how mentors actively compensated for structural limitations by creating relational spaces that promoted accessibility, continuity, and trust.

#### Challenges

Four challenge sub-themes were identified at Site A: time constraints and workload; variable student engagement; managing sensitive or complex issues; and lack of standardisation. All four were mirrored at Site B, with additional specificity. The ‘begging’ metaphor, used independently by multiple Site B participants to describe student disengagement, was a vivid and analytically notable expression of a pattern present at both sites.

*In principle*,* mentorship is useful. But in practice*,* if the students are not interested and the meetings feel compulsory*,* it becomes a ritual rather than a meaningful engagement.* (B-IDI-M9, Site B)

An additional challenge documented at Site B and absent from Site A data was the financial burden on faculty. Mentors reported bearing personal costs for snacks and social outings with no institutional reimbursement, compounding the overall burden of the mentoring role.

*There is no financial support from the institute or government. Even the snacks during meetings are often paid by faculty themselves. That creates an extra financial load too.* (B-IDI-M8, Site B)

The convergence of workload pressures, inconsistent student engagement, and role ambiguity across both institutions indicates that these challenges are embedded within programme implementation rather than being institution-specific problems. Site-specific findings, including financial burden and psychosocial case management, further demonstrate how local organisational contexts shape the additional demands placed on mentors. Collectively, these findings suggest that programme effectiveness depends not only on mentor commitment but also on the extent to which institutional systems support the mentoring role.

#### Recommendations

Site A produced detailed structured recommendations across four sub-themes: a structured monitoring framework including a standard operating procedure, structured logbook, defined schedules, and periodic review, program evaluation; formal training programmes covering communication, mental health counselling, case simulation, and NMC mandate refreshers; recognition and incentivisation through certification, appraisal credit, and cash incentives; and enhanced digital and group communication platforms. Site B recommendations addressed the same structural imperatives with less granular specification, emphasising better programme design, genuine mentor-mentee matching, and institutional administrative backing.

*If mentoring is to continue properly*,* the institution must support it with time*,* structure*,* and some resources. Otherwise it depends too much on the individual faculty member.* (B-IDI-M13, Site B)

Although mentors proposed context-specific recommendations, the underlying pattern across both sites was remarkably consistent. Participants advocated for greater institutionalisation of mentoring through clearer governance, structured monitoring, protected time, faculty development, and administrative recognition. These recommendations collectively indicate that participants viewed mentoring as an organisational responsibility requiring systematic support rather than an activity dependent on individual faculty enthusiasm.

#### Personal reflections

Mentors at Site A documented professional fulfilment, improved communication skills, enhanced empathy and patience, better understanding of the student environment, and the development of a leadership identity as outcomes of the mentoring role. At Site B, these benefits were framed as conditional rather than inherent, arising only when the programme was genuinely organised and supported.

*Mentoring can be useful for both sides. It helps with teaching*,* communication*,* and relationship-building. But that happens only when the process is genuine and supported*,* not when it is just an obligation.* (B-IDI-M11, Site B)

Mentors across both sites recognised personal and professional benefits arising from the mentoring relationship; however, these benefits were perceived as contingent upon meaningful programme implementation. Rather than describing mentoring as inherently rewarding, participants suggested that professional growth emerged when sustained engagement and institutional support enabled authentic mentor–mentee relationships. This finding further reinforces the reciprocal nature of mentoring, whereby both mentor development and student support depend upon favourable organisational conditions.

### Cross-site comparative analysis

#### Integrated challenge matrix

Table 3 presents the integrated challenge matrix drawing on both mentor IDI and mentee FGD findings across both sites. Nine challenge clusters were identified, six of which converged across institutions despite marked differences in geographical setting, programme maturity, and student demographics. This convergence suggests that many implementation barriers are intrinsic to the operationalisation of the NMC-CBME mentorship programme rather than products of individual institutional contexts. Conversely, site-specific challenges—including financial burden on mentors and cultural and language barriers at Site B, and management of complex psychosocial student presentations at Site A—demonstrate how local contextual factors shape the manifestation of these broader structural issues. Together, these findings indicate that mentorship implementation reflects an interaction between common organisational challenges and context-specific adaptations.

**Table 3:** Cross-site challenge matrix: integrated mentor IDI and mentee FGD findings

Challenge	Site A (Jharkhand)	Site B (Arunachal Pradesh)	Source of qualitative data (Site & participant Group)	Cross-site status
Workload overload	Competing academic priorities; limited protected time	*“Mentoring becomes another task squeezed into an already busy schedule”* (B-IDI-M3); *“extra burden”* (B-IDI-M4)	Both	Convergent (high)
Student disengagement	Passive participation; reluctance to share; irregular follow-up	Mentors report *“begging”* students to attend; monosyllabic responses in formal sessions; resigned withdrawal (B-FGD2)	Both	Convergent (high)
Scheduling conflicts	Meeting infrequency (3 to 5 per year); Friday sessions poorly attended; no attendance enforcement	Co-curricular events block scheduling; after-hours and Sunday meeting imposition resented by mentees	Both	Convergent (high)
Role ambiguity and programme artificiality	No monitoring framework; variability in commitment; over-documentation without action	*“Expectations not clear”* (B-IDI-M1); *“ritual rather than meaningful engagement”* (B-IDI-M9); programme-as-checkbox perception	Both	Convergent (high)
Hierarchical communication barriers	Age and seniority barriers; evaluator-role disclosure barrier; power imbalance	Evaluator-role barrier structurally distinct; awkward silences in formal sessions (B-IDI-M8, B-IDI-M6)	Both	Convergent (high)
Weak administrative backing	No standard operating procedure; monitoring framework absent; lack of standardisation	*“Administrative backing is weak”* (B-IDI-M13); no recognition or government support (B-IDI-M12)	Both	Convergent (high)
Financial burden on faculty	Not foregrounded	Faculty pay out-of-pocket for snacks and outings; no institutional reimbursement (B- IDI-M8, B-IDI-M12)	Site B only	Site B-specific
Managing complex student issues	No counselling training; referral uncertainty; emotional burden on mentor	Not foregrounded in Site B transcripts	Site A only	Site A-specific
Cultural and language barriers	Not identified	Cultural inhibition of help-seeking; language medium mismatch in emotional conversations	Site B only	Site B-specific

C = convergent at both sites; A only = Jharkhand-specific; B only = Arunachal Pradesh-specific. Where both mentor and mentee perspectives are available, & the source column refers to the origin of the qualitative data, specifying both the participant group (mentor IDIs or mentee FGDs) and the study site (Site A or Site B)

#### Communication modalities across sites

**Fig. 2:**
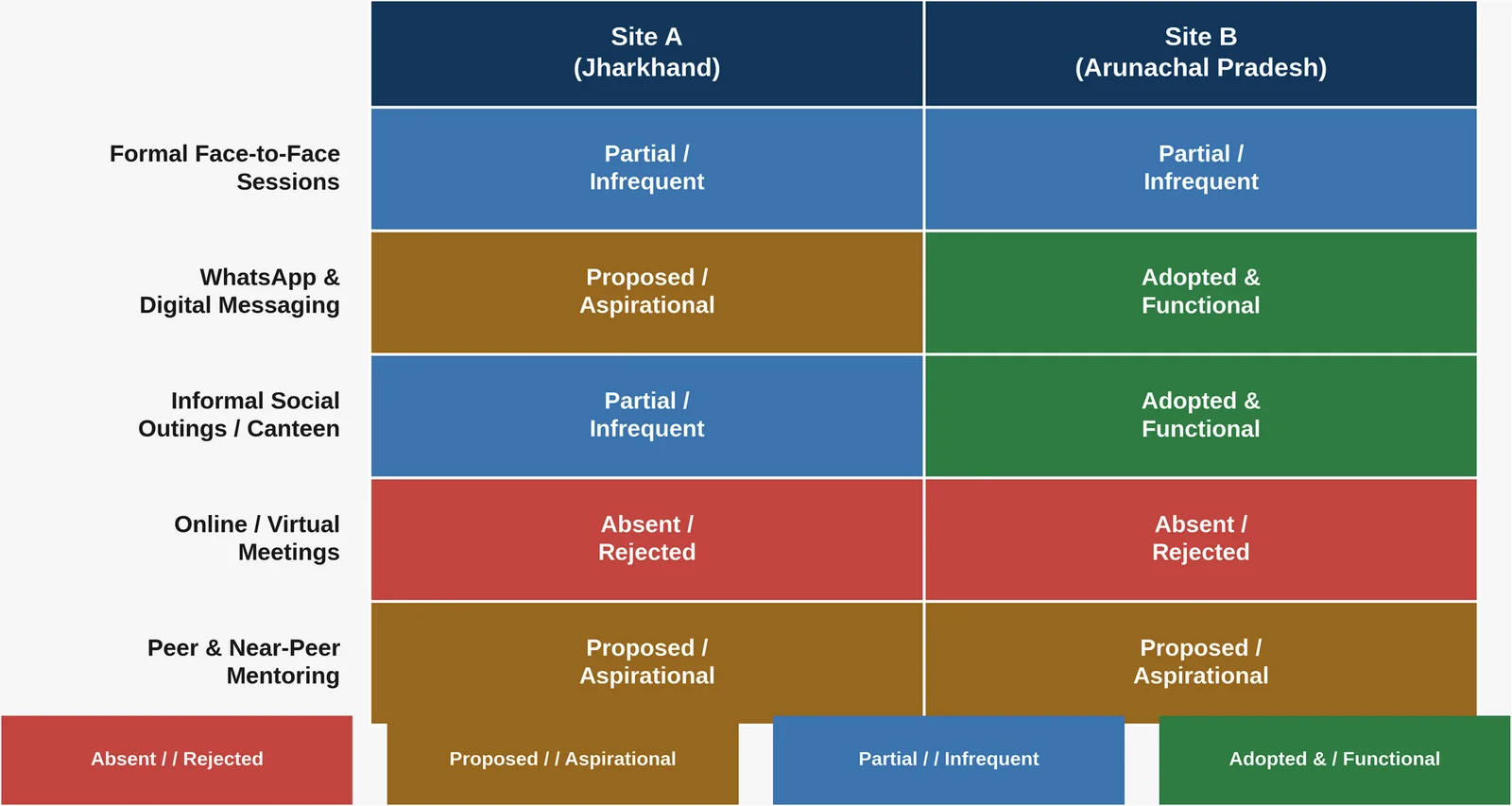
Communication modalities across sites: mentor and mentee perspectives. Site B has independently adopted strategies (WhatsApp, social outings) that Site A is still recommending as future solutions

The communication modalities documented across both sites from both mentor and mentee perspectives were compared in (Fig. 2). The comparison of communication modalities revealed a noteworthy pattern across both institutions. Site B mentors independently developed informal approaches, including WhatsApp communication and social interactions, as practical adaptations to limited formal meeting opportunities. Interestingly, participants at Site A proposed many of these same approaches as desirable future improvements. This convergence suggests that relational continuity may be achieved through flexible communication strategies when institutional structures are insufficient, although such approaches should be implemented with appropriate consideration of professional boundaries, confidentiality, and institutional guidance. Rather than representing isolated innovations, these findings illustrate how local adaptations may inform future programme refinement across comparable settings.

#### Cross-Site Comparison: Mentor IDIs

Table 4 presents the comparative analysis of mentor IDI findings across both sites. The pattern of convergence on structural challenges, including workload burden, student disengagement, and role ambiguity, and partial convergence on adaptive strategies and personal benefits is consistent with the convergence observed in mentee FGD data.

The high degree of convergence in mentor experiences across two organisationally distinct institutions strengthens confidence that these findings represent recurring implementation patterns rather than isolated institutional phenomena. At the same time, differences in adaptive strategies highlight the influence of local organisational culture on how mentors negotiate common structural constraints. These findings reinforce the importance of preserving contextual sensitivity while recognising shared priorities for programme improvement.

**Table 4:** Cross-site comparison of mentor IDI findings

Domain/Theme	Site A (Jharkhand)	Site B (Arunachal Pradesh)	Status	Key finding
Workload and time burden	Competing academic priorities; no protected time; scheduling a sub-theme in Challenges	*“Mentoring becomes another task squeezed into an already busy schedule”* (B-IDI-M3); extra burden (B-IDI-M4)	C	Protected time universally absent at both sites
Student disengagement	Passive participation; irregular follow-up; reluctance to share	*“Feels like begging students to come”* (B-IDI-M1, B-IDI-M4); monosyllabic responses (B-IDI-M6, B-IDI-M8)	C	Vivid *“begging”* metaphor specific to Site B; underlying pattern convergent
Role ambiguity	Variable commitment; monitoring absent; over-documentation	*“Expectations not clear”* (B-IDI-M1); programme felt forced (B-IDI-M4, B-IDI-M9); *“not supposed to be close friends”* (B-IDI-M5, B-IDI-M9)	C	Both sites report role confusion; Site B more explicit about social awkwardness of imposed closeness
Managing complex student issues	No counselling training; referral uncertainty; emotional burden	Not foregrounded in Site B IDI transcripts	A only	Reported at Site A; not foregrounded in Site B IDI transcripts
Financial burden	Not foregrounded	Faculty pay out-of-pocket for snacks and outings; no government support (B-IDI-M8, B-IDI-M12)	B only	Site B-specific; absence of institutional reimbursement documented
Informal engagement as adaptive strategy	Digital platforms recommended but not yet implemented; group sessions proposed	WhatsApp organically adopted (B-IDI-M3, B-IDI-M10, B-IDI-M6); social outings improve bonding (B-IDI-M7)	P	Site B has adopted what Site A is still proposing; cross-site learning opportunity
Personal benefits to mentors	Professional fulfilment; improved communication, empathy, patience; leadership identity	Benefits conditional: *“only when genuine and supported”* (B-IDI-M11); confidence gains (B-IDI-M14)	P	Site A reports richer articulation of personal growth; Site B frames benefits as contingent on programme quality
Recommendations: structural	Standard operating procedure, logbook, defined schedules, periodic review	Better programme design and matching (B-IDI-M10); institution must support with time, structure, and resources (B-IDI-M13)	C	Both sites demand structural reform; Site A more granular in specification
Recognition and incentives	Certification, appraisal credit, cash incentives, curriculum inclusion	Not explicitly articulated; implied through burden narrative	A only*	Raised explicitly at Site A; not foregrounded in Site B IDI transcripts

*C *convergent, *P * partially convergent, *A *only  Jharkhand-specific, *B* only  Arunachal Pradesh-specific. *Recognition and incentives: not foregrounded in Site B IDI transcripts

### Student-generated recommendations

Table 5 presents recommendations generated directly by student participants across both sites, with corresponding mentor corroboration from IDI findings. The specificity and internal consistency of these recommendations, generated independently by participants at two geographically distinct sites, strengthens their validity as priority targets for programme reform.

**Table 5:** Student-generated recommendations with mentor corroboration

Construct	Recommendation (student-expressed)	Source site	Mentor corroboration
Programme orientation	A formal orientation at the start of first year communicating programme goals, student rights, and expected meeting frequency to all new students and their mentors	Both sites	Yes (both sites)
Scheduling: protected time	A designated and protected meeting slot within the institutional timetable, within working hours, non-optional for both parties	Site B primary	Yes (both sites)
Scheduling: standardised venue	Meetings standardised and held in a common venue such as a lecture theatre to ensure uniformity and accessibility	Site A	Yes (both sites)
Mentor training	Formal mentor training covering structured questioning, active listening, and proactive academic monitoring	Site B primary	Yes (both sites)
Documentation and continuity	A mandatory logbook recording meeting content, academic progress, and agreed actions, accessible to both parties and persistent across mentor transitions	Site B	Yes (Site A explicit)
Student feedback mechanism	An anonymous mid-year feedback mechanism allowing students to report on programme quality without fear of identification	Site B	Partial
Mentor recognition	Formal acknowledgment of mentoring quality, including appraisal credit and certificates, making good mentoring institutionally rewarded rather than dependent on voluntary commitment	Site B primary	Yes (Site A explicit)
Confidentiality policy	An explicit and communicated confidentiality policy clarifying what is and is not reported, to reduce self-censorship in personal disclosure	Site B	Not raised by mentors
Phase-appropriate structure	Intensive structured mentoring in the first three months, with a more flexible, needs-based approach in subsequent years	Site A	Partial (both sites)
Near-peer mentoring integration	Integration of senior students in an informal near-peer advisory capacity alongside the formal faculty mentoring relationship	Both sites (minority at Site B)	Site A IDI (group sessions)
Mentor attendance accountability	Attendance marking or equivalent mechanism to ensure formal commitment from mentors to scheduled sessions	Site A	Partial
Response time standard	A defined maximum response time for mentor replies to student messages, with an institutional escalation pathway if unmet	Site B	Not raised by mentors

Recommendations are derived from FGD data at both sites. Mentor corroboration is derived from IDI findings. “Both sites” indicates the recommendation was expressed independently at both Arunachal Pradesh and Jharkhand

The close correspondence between student recommendations and mentor perspectives provides evidence of substantial stakeholder agreement regarding priorities for programme reform. Rather than reflecting competing expectations, both groups consistently identified organisational improvements—including structured scheduling, clearer communication pathways, enhanced mentor preparation, and systematic evaluation—as central to improving mentoring effectiveness. This convergence strengthens the practical relevance of these recommendations for future programme development.

### Overall convergence summary

**Fig. 3:**
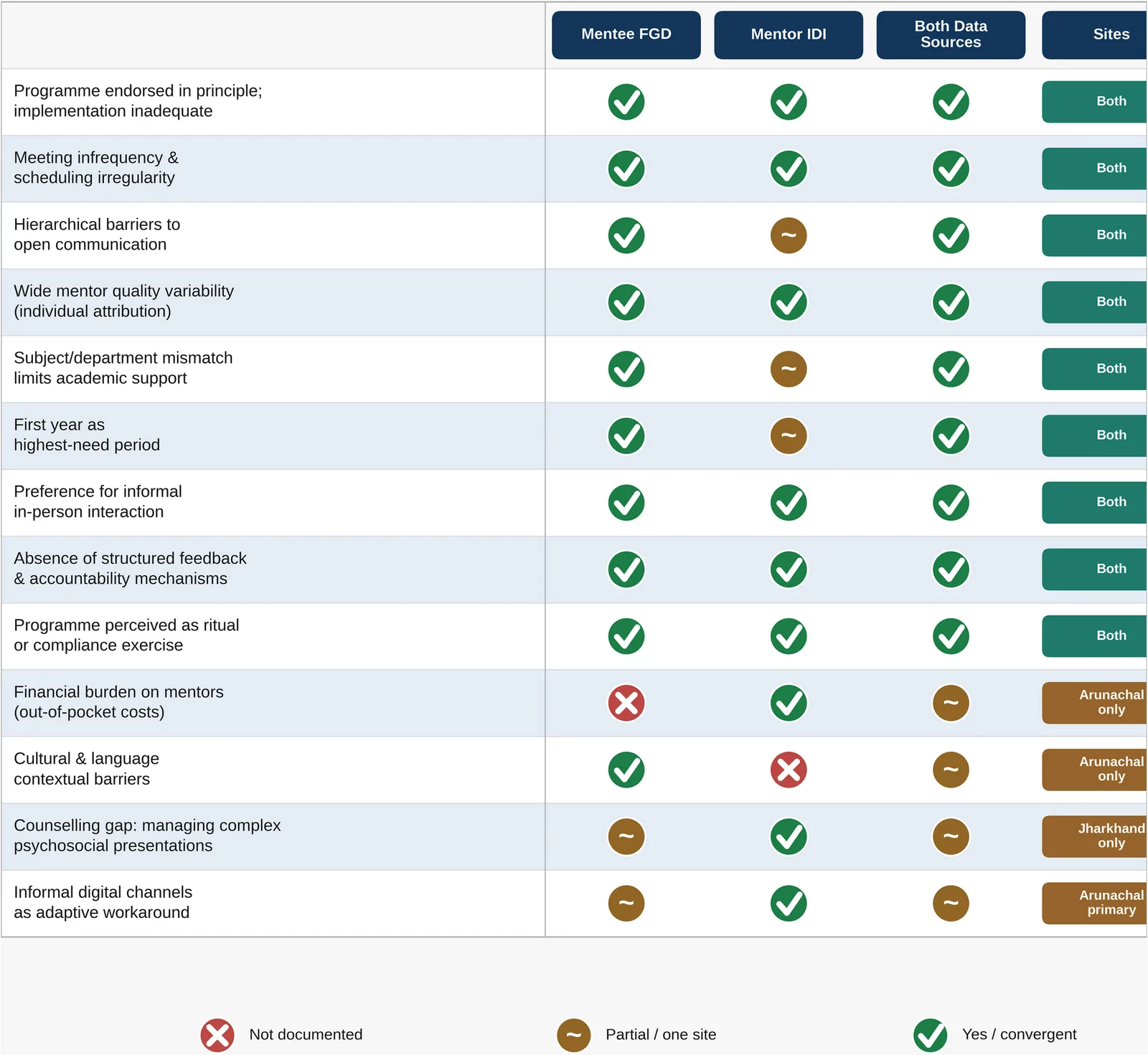
Overall cross-source, cross-site convergence summary. The most consistent pattern across all four data sources is that the programme is endorsed in principle while implementation is experienced as inadequate

A consolidated cross-source, cross-site summary integrating mentor IDI and mentee FGD data from both sites was presented in the (Fig. 3). Across all four data sources, the most consistent pattern is that the programme is endorsed in principle while its implementation is experienced as structurally inadequate.

Across mentor and mentee datasets, and across both institutions, a coherent interpretive pattern emerged. Participants consistently endorsed the educational value and intended purpose of the NMC-CBME mentorship programme; however, they perceived that its implementation was constrained by organisational factors including limited protected time, role ambiguity, inadequate mentor preparation, inconsistent scheduling, and the absence of systematic programme monitoring. Consequently, the quality of mentoring relationships depended largely on individual mentor initiative and locally developed adaptive practices rather than consistently supported institutional processes. Despite substantial contextual differences between the two institutions, the convergence of these findings suggests that many implementation challenges are shared across settings, whereas contextual differences primarily influenced how participants adapted to these structural constraints. Collectively, these findings indicate that strengthening organisational systems may have greater impact on programme effectiveness than focusing solely on individual mentor or mentee behaviours.

## Discussion

This two-centre qualitative study of the NMC-CBME mentorship programme is, to our knowledge, among the first to compare mentor and student experiences of this programme systematically across geographically and demographically distinct institutions in India. The existing literature on CBME mentorship in Indian settings has tended to focus on single institutions, often using primarily quantitative measures of meeting frequency or student satisfaction, without capturing the relational and structural texture of how the programme is experienced by both parties [11, 12]. 

Collectively, these design features enabled the identification of patterned meanings that extended beyond individual participant accounts, allowing organisational and contextual influences on mentorship implementation to be interpreted across diverse institutional settings. This study contributes in three specific respects. First, it provides parallel accounts from both mentors and students within the same institutional settings, allowing the convergence and divergence of their perspectives to be examined rather than assumed. Second, the two-site design enables a form of comparative analysis where similar findings across dissimilar institutions carry stronger interpretive weight than they would from a single site. Third, the contextual findings from Site B, particularly the identification of cultural inhibition of help-seeking, language medium mismatch, and the financial burden on faculty, introduce considerations that are absent from most prior literature on this programme and that are directly relevant to programme implementation in tribal and minority student populations across northeastern India.

The convergence of challenge profiles across two institutions separated by geography, administrative culture, and student demographics is the most analytically informative pattern in the data. Rather than representing isolated institutional shortcomings, this convergence suggests that organisational structures exert a greater influence on mentoring experiences than individual mentor or student characteristics, a pattern that was consistently observed across both stakeholder groups. Across both sites and both data sources, a consistent set of structural difficulties emerges: workload compression, scheduling infrequency, role ambiguity, hierarchical communication barriers, and the narrowing of mentorship activity to documentation and meeting counts rather than relational engagement. This convergence pattern is consistent with broader evidence from qualitative studies of formal mentorship programmes in medical education, which have identified structural inadequacy, rather than interpersonal failure, as the primary determinant of programme effectiveness [15, 16]. 

The tension between documentation-driven compliance and relational engagement that emerged at both sites reflects a well-documented problem in educational mentorship literature: the distinction between compliance-oriented and relationship-oriented programme designs [17]. When programmes are designed primarily around documentation and meeting counts, participants adapt to those targets rather than to relational goals. The NMC mandate, as currently operationalised, appears to have generated compliance infrastructure without the relational infrastructure required to make compliance meaningful. Sustainable mentoring cultures require institutional embedding, not merely individual commitment; programmes dependent on exceptional individual motivation are inherently fragile [18]. 

The evaluator-mentor role conflict identified by students at Site B is a finding with clear design implications, though its magnitude in practice is likely to vary by institutional culture and how clearly programme boundaries are communicated to participants. Where the same person grades internal assessments and provides specialist references, the conditions required for genuine personal disclosure are structurally at risk. This dual-role vulnerability has been documented in international medical education literature, where it has been associated with strategic rather than authentic student engagement [19, 20]. Solutions proposed in the literature include role separation through assignment of non-evaluating mentors, or the implementation of explicit, institutionally endorsed confidentiality protocols that clarify the boundaries of disclosure [19, 21]. Either approach requires deliberate programme design rather than reliance on individual faculty judgment.

The trust formation findings at both sites align with theoretical frameworks of relational mentoring that conceptualise trust as a developmental achievement rather than a baseline condition [22]. Students who met their mentors three to five times per year were reporting the absence of a condition, a trusting relationship, that takes sustained, consistent engagement to develop. The frequency deficit identified in this study is consistent with findings from Indian medical education literature, where even mandated programmes have been shown to operate well below their stated meeting frequencies [11, 12]. 

The informal adaptive strategies documented at Site B, including WhatsApp-based communication and social outings, represent a notable cross-site observation. The same strategies are proposed as future recommendations by Site A mentors, which at minimum suggests they address a real gap rather than being incidental innovations.

Although participants described WhatsApp as a convenient medium for maintaining regular mentor–mentee communication, its use should be accompanied by appropriate institutional guidance to ensure confidentiality, privacy, and professional boundaries. Digital communication platforms may enhance accessibility and continuity of mentoring; however, their implementation should align with institutional data protection policies and ethical standards governing professional communication.

Evidence from medical education settings indicates that instant messaging tools can reduce communicative barriers between students and faculty and extend mentoring contact beyond the constraints of formal scheduled meetings, though their effectiveness depends on how they are integrated into an institutional framework rather than left to individual initiative [23]. The financial burden on faculty for social outings, documented at Site B, represents an inequity that would need to be addressed institutionally before these practices could be adopted equitably elsewhere.

The phase-specific mentorship needs identified at Site A, and partially supported at Site B, are consistent with the developmental stage literature on medical student formation [24]. The first three months of MBBS represent the period of greatest transition from secondary education to the demands of a professional curriculum, and the findings confirm that this period commands the greatest mentorship need. The explicit three-phase model articulated by Site A students, specifically intensive first year, autonomous middle years, and career-focused final year, provides a practical template for phase-sensitive programme redesign.

The cultural and contextual findings from Site B constitute a distinctive contribution of this study. The clarification that shared tribal identity is less relevant than contextual literacy, understood as an understanding of the social and material conditions of students from Arunachal Pradesh, is consistent with qualitative evidence showing that what minority students most need from mentors is not demographic concordance but responsiveness to their specific social and institutional circumstances, including recognition of cultural communication norms and the particular barriers to help-seeking that their backgrounds create [25]. This reframes the challenge at Site B as one of mentor training and orientation rather than demographic matching, and it points to interventions that are practically achievable without changes to the faculty composition of the institution.

The language medium mismatch finding, though arising from a single participant account, is consistent with evidence on the role of language in therapeutic and support relationships, where emotional resonance depends on shared communicative registers [26]. Mentor cultural orientation covering regional communication norms and the avoidance of Hindi idioms in emotionally sensitive conversations would represent a targeted, low-cost intervention at Site B and in similar institutional contexts across India.

The data also contain substantive positive findings that should not be obscured by the dominant challenge narrative. At both sites, a number of mentor-mentee relationships were described in terms that went well beyond procedural compliance: mentors who identified learning styles, provided career networking, disclosed their own academic struggles, or engaged families were experienced as genuinely impactful by students. Site A mentors articulated meaningful personal benefits from the role, including enhanced communication skills, empathy, and a developing sense of professional identity. At Site B, informal social outings produced the most warmly described relational moments in the data. The picture that emerges is not one of a fundamentally flawed programme but of one whose positive potential is realised when individual conditions align and structural supports are in place, and constrained when they are not. Taken together, these findings suggest that the effectiveness of mentorship is not determined solely by programme design or individual mentor characteristics but by the interaction between organisational support, relational continuity, and locally adaptive practices. The consistency of this pattern across two markedly different institutional contexts strengthens the interpretation that sustainable mentoring depends on institutional systems that enable meaningful relationships rather than relying primarily on individual commitment.This distinction matters for how reform is framed: the goal is to extend the conditions that produce good mentoring, not to redesign from scratch.

The student-generated recommendations in that are notable for their specificity and convergence with international evidence on effective mentorship programme design. Protected meeting time, structured mentor training, explicit confidentiality protocols, anonymous feedback mechanisms, and recognition of good mentoring through appraisal credit are recommendations that appear consistently in mentoring literature across medical and health professional education settings [15, 16]. Their independent generation by student participants at two geographically distinct sites strengthens their validity as priority targets for programme reform.

## Limitations

This study has several limitations. First, participants at both sites were recruited through purposive sampling to capture diverse mentor and mentee perspectives; however, this approach may have introduced selection bias and limits statistical representativeness. The findings are therefore intended to support analytical transferability rather than statistical generalisation. Although participation was voluntary and all participants were explicitly informed that declining participation would have no academic or professional consequences, the hierarchical nature of medical education means that some students or faculty may nevertheless have perceived an obligation to participate or may have moderated their responses. Similarly, focus group discussions are inherently susceptible to social desirability bias and group dynamics, despite facilitation strategies and concurrent documentation of group interactions by a research assistant to encourage balanced participation.

Second, no formal member checking or participant validation was undertaken, which may have limited opportunities for participants to confirm the researchers’ interpretations. Although an audit trail, independent coding by two analysts, consensus discussions, and iterative refinement of themes were employed to enhance trustworthiness, the findings remain influenced by the interpretive lens of the research team. Furthermore, while the investigators were not participants in the mentorship programme, their institutional and disciplinary backgrounds may have influenced data collection and interpretation despite the reflexive measures undertaken.

Third, participant demographic characteristics were intentionally reported at a summary level to preserve confidentiality; however, more detailed demographic information may have assisted readers in assessing the transferability of the findings. Interviews and focus group discussions were conducted primarily in English with occasional use of Hindi and local language expressions. Although transcripts were translated and verified where required, subtle linguistic and cultural nuances may not have been captured completely.

Finally, the study was conducted in only two purposively selected medical colleges that differed substantially in geography, student demographics, programme maturity, and institutional resources. While this contextual diversity strengthens analytical comparison, the non-equivalence of sites precludes causal interpretation of observed differences. Consequently, the findings should be considered analytically transferable rather than statistically generalisable and interpreted within the context of similar educational settings. As the study reflects experiences during a specific phase of implementation of the NMC-CBME mentorship programme, the findings may not fully represent subsequent programme modifications following institutional or regulatory changes.

## Conclusion

This two-centre qualitative study explored the experiences of mentors and mentees participating in the NMC-CBME mentorship programme across two geographically and institutionally distinct medical colleges in India. Both stakeholder groups recognised the value of mentorship for academic, professional, and personal development; however, they consistently described implementation challenges related to workload, limited protected time, role ambiguity, hierarchical communication, and inadequate institutional support. While the specific manifestations of these challenges varied across sites, substantial convergence was observed despite differences in programme maturity, institutional context, and student demographics, suggesting that many implementation issues may extend beyond individual institutions.

The findings also highlight the influence of contextual factors, including cultural communication norms, language, and locally developed adaptive practices, in shaping mentorship experiences. Together, these findings suggest that effective implementation of the NMC-CBME mentorship programme requires context-sensitive institutional support, in terms of protected and timetabled meeting time; structured, competency-based mentor training; explicit confidentiality protocols that separate the developmental from the evaluative role; institutional recognition of good mentoring through appraisal and certification, structured mentor development, mechanisms for continuous programme evaluation, and attention to the quality of mentor–mentee relationships though feedback mechanisms. It also indicates that generic programme templates are insufficient for the diverse institutional settings in which medical education is delivered across India, and that context-sensitive adaptation, covering cultural communication norms, help-seeking inhibition, and language medium, is necessary for effective programme implementation in minority and tribal student populations. As this study was conducted in two purposively selected institutions, these conclusions should be interpreted as analytically transferable insights that warrant further evaluation across a broader range of medical colleges.

## Supplementary Information

Supplementary Material 1.

Supplementary Material 2.

Supplementary Material 3.

## Data Availability

Data are not publicly available due to ethical restrictions and participant confidentiality. De-identified data may be made available from the corresponding author on reasonable request, subject to ethical oversight.

## Abbreviations

CBME: Competency-Based Medical Education
NMC: National Medical Commission
FGD: Focus Group Discussion
IDI: In-Depth Interview
MBBS: Bachelor of Medicine and Bachelor of Surgery
IEC: Institutional Ethics Committee
COREQ: Consolidated Criteria for Reporting Qualitative Research
AS: Amrita Sarkar
JB: Jarina Begum
DR: Debjit Roy
SS: Swati Shikha
SIA: Syed Irfan Ali
