# Supplementary material for: Mentor and mentee experiences of the NMC-CBME mentorship programme in India: a two-centre qualitative study
Source: BMC Med Educ. 2026 Aug 11;26:1800. doi: 10.1186/s12909-026-10132-x (PMC13741466; doi:10.1186/s12909-026-10132-x)
Supplement: Supplementary file 1 [file 12909_2026_10132_MOESM1_ESM.pdf]

<u>Qualitative Data Collection Tools:</u> (FGDs & IDIs)

Guide for Focus Group Discussion (Approx 75-100 Minutes)

Welcome, mentees, to this focus group discussion aimed at exploring your experiences and perceptions of the mentorship program. Your insights are crucial in understanding the impact of mentorship on your personal and professional development. We appreciate your willingness to share your thoughts openly.

Introductions and Icebreaker

1.1 Introductions

1. Please introduce yourself, sharing your name, and your current year of study.

Mentorship Experience

2.1 **Matching Process and Initial Impressions**

2. How did the mentor-mentee matching process take place?

3. What were your initial impressions of your mentor?

4. Was there a specific goal or expectation you had in mind when you started?

2.2 Relationship Dynamics

a) Describe the nature of your relationship with your mentor. How would you characterise your interactions?

b) Has the mentorship relationship met your expectations so far?

Can you provide examples to illustrate its impact on your development?

2.3 Learning and Skill Development

a) In what ways has the mentorship program contributed to your learning and skill development?

Have there been any specific instances where your mentor's guidance enhanced your understanding or abilities?

b) How has your mentor helped you navigate challenges or obstacles in your medical education journey?

Challenges and Barriers

3.1 Communication and Availability

3. Have you encountered any challenges in terms of communication with your mentor?

4. How responsive and available has your mentor been in addressing your concerns or queries?

3.2 Balancing Expectations and Personal Goals

a) Have you experienced any conflicts or discrepancies between your personal goals and the expectations set within the mentorship program?

How have you managed to address or reconcile them?

b) In what ways has your mentor supported you in aligning your goals with the requirements of your medical education?

Recommendations and Suggestions

4.1 Program Improvement

4. Based on your experience, what specific improvements or changes would you suggest to enhance the mentorship program and make it more beneficial for mentees like yourself?

4.2 Mentor Support and Training

a) Do you feel your mentor has received sufficient training to fulfil their role effectively? Are there any areas where you think additional training or support would be beneficial?

b) How can the mentorship program provide better guidance or resources to mentors, enabling them to support mentees more effectively?

Closing Remarks

5.1 Key Learnings and Growth

5. Reflecting on your mentorship journey, what are the key learnings or moments of personal growth you have experienced? Please share any specific insights that have positively influenced your development.

5.2 Appreciation

We sincerely appreciate your active participation in this focus group discussion. Your input will significantly contribute to improving the mentorship program and enhancing the experiences of future mentees. Thank you for your valuable contribution.

Guide for In-Depth Interview (45-60 Minutes) :

An interview guide to ask mentors about their needs, strengths, challenges, and recommendations:

1. Introduction:

- Thank the mentor for their participation in the mentorship program.
- Briefly explain the purpose of the interview: to gather their insights on their experience as a mentor and gather feedback to improve the program.

2. Mentor's Needs:

- What are your specific needs or expectations as a mentor in this program?

- Are there any resources, support, or training that you feel would enhance your effectiveness as a mentor?
- How can the program better support you in fulfilling your role as a mentor?

3. **Mentor's Strengths:**

- What strengths or skills do you bring to the mentorship program?
- In what areas do you feel most confident in supporting your mentees?
- Can you provide examples of how your strengths have positively impacted your mentees' development?

4. **Mentor's Challenges:**

- Have you encountered any challenges or obstacles in your role as a mentor? If yes, could you please describe them?
- How have you addressed these challenges and what strategies have you found effective?
- Is there any specific support or guidance you would like to receive to overcome these challenges?

5. **Program Recommendations:**

- Based on your experience, what recommendations or suggestions do you have for improving the mentorship program?
- Are there any additional resources, training, or activities you believe would benefit both mentors and mentees?
- How can the program better facilitate effective communication and collaboration between mentors and mentees?

6. **Mentor's Personal Reflection:**

- How has being a mentor in this program impacted you personally?
- Have you experienced any personal growth or development through your role as a mentor?
- Are there any specific rewards or fulfilment you derive from being a mentor?

7. **Conclusion:**

- Thank the mentor for their valuable insights and time.
- Express appreciation for their dedication to the mentorship program and its impact on the mentees.
- Assure them that their feedback will be taken into consideration to enhance the program.
