# Supplementary material for: Mentor and mentee experiences of the NMC-CBME mentorship programme in India: a two-centre qualitative study
Source: BMC Med Educ. 2026 Aug 11;26:1800. doi: 10.1186/s12909-026-10132-x (PMC13741466; doi:10.1186/s12909-026-10132-x)
Supplement: Supplementary file 3 [file 12909_2026_10132_MOESM3_ESM.pdf]

Part A. Mentee Focus Group Discussion (FGD) Data

Nine provisional themes had emerged from inductive thematic analysis of the FGD transcripts at the point this draft was prepared. Eight were convergent across both sites; one was identified only at Site B and was still under discussion as to whether it should stand as a separate, site-specific theme (resolved in Round 2, see T9 below).

T1.

Provisional theme grouping open codes on the mismatch between the programme as designed and as delivered.

Working code / category (Round 1, independent coding)	Reconciliation note (Round 2 / Round 3)	Site(s)	Provisional mapping (subject to finalisation)
'no one contacted me' / uninitiated contact	Confirmed by both coders independently (Site B); retained without relabelling	Site B	T1 · Programmatic invisibility
'nobody checks attendance' / attendance not enforced	Confirmed Round 1; wording tightened Round 2 from initial label 'lax attendance'	Site A	T1 · Scheduling irregularity / non-enforced attendance
oriented vs unoriented batch contrast	Flagged Round 1 by one coder only; confirmed as a genuine cross-batch pattern after second coder re-checked B-FGD1 against B-FGD2 to 4 in Round 2	Site B	T1 · Orientation as institutional legitimation (cross-batch contrast)

T2.

Two coders initially split this into separate frequency and boundary categories; merged into one theme with two components in Round 2.

Working code / category (Round 1, independent coding)	Reconciliation note (Round 2 / Round 3)	Site(s)	Provisional mapping (subject to finalisation)

'meetings do not happen regularly' / low meeting frequency	Confirmed both sites; structural attribution language added in Round 2 after team discussion of whether to frame as individual or systemic	Both sites	T2 · Sub-standard meeting frequency (structural attribution)
Sunday meeting complaint	Initially coded as a single-participant grievance; escalated for discussion after second coder felt it reflected a broader boundary issue; resolved by consensus to retain as distinct category	Site B	T2 · Sunday / after-hours scheduling as boundary violation
protected slot / fixed venue suggestion	Independently proposed at both sites using different wording ('timetabled slot' vs 'common venue'); reconciled into a single cross-site category in Round 2	Both sites	T2 · Structural solution proposal (protected slot / standardised venue)

T3.

Working label 'mentor effort variation' in Round 1; renamed after Round 2 to foreground the mechanism (proactivity) rather than the outcome.

**Working code / category (Round 1, independent coding)**	**Reconciliation note (Round 2 / Round 3)**	**Site(s)**	**Provisional mapping (subject to finalisation)**
proactive, individualised academic advice	Confirmed Round 1	Site B	T3 · Mentor proactivity generating accountability
generic 'talk to your teachers' advice	Confirmed Round 1; kept as the low-effort counterpart to the category above	Site A	T3 · Generic / non-specific academic advice
faculty sharing own academic struggles	Noted narratively rather than as a standalone excerpt; flagged for third-reviewer check on whether it stood alone as a category or belonged inside proactivity; retained separately by consensus	Site B	T3 · Faculty self-disclosure of academic struggle

mentor as networking / referral bridge	Confirmed Round 1	Site B	T3 · Career guidance / networking access (Phase III)
specialty mismatch limiting relevance	Identified independently at both sites in Round 1; treated as convergent without further discussion	Both sites	T3 · Specialty-phase mismatch (structural constraint)

T4.

Largest cluster of open codes in Round 1 (six candidate codes); condensed to four categories after Round 2 merged two closely overlapping codes on confidentiality.

Working code / category (Round 1, independent coding)	Reconciliation note (Round 2 / Round 3)	Site(s)	Provisional mapping (subject to finalisation)
mentor-as-examiner conflict of interest	Confirmed Round 1	Site B	T4 · Evaluator-mentor role conflict / self-censorship
seniority / age hierarchy inhibits disclosure	Confirmed Round 1	Site A	T4 · Age hierarchy / administrative seniority inhibition
mentor involving family (parental contact)	Single-transcript origin; discussed in Round 2 as to whether it generalised; retained as a distinctive contextual adaptation rather than removed	Site A	T4 · Parental engagement by mentor (distinctive contextual adaptation)
'cannot open up without knowing them' / trust needs time	Confirmed both sites	Both sites	T4 · Trust as a developmental outcome requiring sustained contact
uncertainty over what stays confidential (two overlapping open codes)	Round 1: two coders logged this separately as 'confidentiality worry' and 'disclosure risk'; merged into a single category in Round 2 after discussion showed they described the same phenomenon	Site B	T4 · Confidentiality ambiguity

T5.

Confirmed at both sites in Round 1; framing debated in Round 2 (individual failing versus structural absence of preparation), resolved by consensus to present both.

Working code / category (Round 1, independent coding)	Reconciliation note (Round 2 / Round 3)	Site(s)	Provisional mapping (subject to finalisation)
mentor was absent / unreachable	Confirmed Round 1	Site A	T5 · Mentor absence / disengagement
sessions drift into social chat, no agenda	Attributed to absence of structured guidance/training rather than personal disinterest after team discussion in Round 2	Both sites	T5 · Absence of structured questions, sessions default to social exchange
good mentors are the exception, not the norm	Confirmed both sites	Both sites	T5 · Outcome variability tied to individual initiative rather than structural preparation

T6.

Two distinct sub-barriers identified in Round 1 and kept separate rather than merged, since one is evaluator-specific and the other is generic seniority.

Working code / category (Round 1, independent coding)	Reconciliation note (Round 2 / Round 3)	Site(s)	Provisional mapping (subject to finalisation)
nervous / power imbalance because mentor also evaluates	Confirmed Round 1	Site B	T6 · Evaluator-role communication barrier (structurally distinct)
hesitant to speak openly with senior / HOD mentor	Confirmed Round 1	Site A	T6 · Age / departmental seniority barrier

T7.

Provisionally labelled 'timing of support' in Round 1; only Site A data consolidated cleanly into a three-phase model, flagged for Discussion-section caveat rather than forcing symmetry with Site B.

Working code / category (Round 1, independent coding)	Reconciliation note (Round 2 / Round 3)	Site(s)	Provisional mapping (subject to finalisation)
early months hardest / need most support then	Confirmed both sites	Both sites	T7 · First-year / initial-months high-need period
want postgraduate / career guidance closer to finals	Confirmed Round 1	Site A	T7 · Final-year career / postgraduate-guidance need
phase variation present but not organised into stages	Round 2 discussion on whether to force a matching three-phase structure at Site B; decided against it, noted as a genuine cross-site difference rather than a coding gap	Site B	T7 · Phase-specific variation present but not consolidated into a distinct sub-framework

T8.

Most heavily revised theme: three overlapping Round 1 codes at Site B collapsed into two categories, with a third, cross-source category added after Round 3 cross-checking against mentor and administrator data.

Working code / category (Round 1, independent coding)	Reconciliation note (Round 2 / Round 3)	Site(s)	Provisional mapping (subject to finalisation)
only meeting counts are tracked, not content or benefit	Confirmed Site B Round 1; Site A version derived indirectly through standardisation recommendations rather than a direct excerpt	Site B (developed); Site A (via recommendations)	T8 · Absence of student feedback mechanism
no one measures whether the programme works	Initially logged as part of the feedback-mechanism code; split out in Round 2 as conceptually distinct (process tracking vs outcome measurement)	Site B	T8 · Non-measurement of programme outcomes
programme treated as a compliance / tick-box exercise	Not present as a standalone code until Round 3, when cross-checking against Figure 3 (all four data sources) showed convergence; added at that stage	Site B; corroborated cross-source	T8 · Institutional relegation of the programme to a compliance exercise

T9.

Confirmed as Site-B-unique in Round 1; team discussed in Round 2 whether to fold into existing themes or retain as a ninth, site-specific theme, decided on the latter to avoid diluting a genuinely distinct pattern.

**Working code / category (Round 1, independent coding)**	**Reconciliation note (Round 2 / Round 3)**	**Site(s)**	**Provisional mapping (subject to finalisation)**
competence over shared tribal identity as the trust criterion	Confirmed Round 1	Site B	T9 · Contextual literacy valued over shared tribal identity
hesitation to discuss mental health due to hierarchy/face	Confirmed Round 1	Site B	T9 · Cultural coding of disclosure as weakness / loss of face
language or idiom mismatch in an emotional conversation	Single-participant account; flagged for cautious interpretation in the Discussion rather than removed	Site B	T9 · Language / idiom mismatch in an emotionally significant conversation

Part B. Mentor In-Depth Interview (IDI) Data

Site A interviews were analysed using framework analysis combined with inductive thematic analysis, with the five domains below functioning as the framework matrix headers from the outset. Site B interviews were analysed using open inductive coding; the resulting codes were aligned to the same five domain headings during Round 2 to allow cross-site comparison, as reflected in the reconciliation notes below.

D1.

Site A organised through the framework matrix from the outset; Site B began as open inductive codes and was aligned to the same domain heading in Round 2.

**Working code / category (Round 1, independent coding)**	**Reconciliation note (Round 2 / Round 3)**	**Site(s)**	**Provisional mapping (subject to finalisation)**

wants a clear orientation to the role	Aggregated pattern, no standalone excerpt; confirmed via coded transcript summary	Site A	D1 · Structured orientation (role and responsibilities)
wants training in communication, active listening, conflict handling	Aggregated pattern, no standalone excerpt	Site A	D1 · Faculty development programme
wants protected time and institutional recognition	Aggregated pattern, no standalone excerpt	Site A	D1 · Institutional support
'expectations are not very clear'	Confirmed Round 1, retained verbatim	Site B	D1 · Clarity of expectations
needs training, time, and support	Confirmed Round 1	Site B	D1 · Training, time, and support required
unclear scope and no allocated time, treated as the primary constraints	Elevated in Round 2 from a general complaint to a named category after recurring across multiple Site B interviews	Site B	D1 · Unclear scope and absence of time allocation as primary constraints

D2.

Confirmed independently at both sites with limited overlap in specific content, reflecting the different coding approaches used.

**Working code / category (Round 1, independent coding)**	**Reconciliation note (Round 2 / Round 3)**	**Site(s)**	**Provisional mapping (subject to finalisation)**
builds interpersonal and communication skills (mentor side)	Aggregated pattern, no standalone excerpt	Site A	D2 · Interpersonal and communication skills
opportunity for professional role modelling	Aggregated pattern, no standalone excerpt	Site A	D2 · Professional role modelling
supports the whole student, not just academics	Aggregated pattern, no standalone excerpt	Site A	D2 · Holistic student support
informal outings (meals, films) improve bonding	Confirmed Round 1	Site B	D2 · Informal bonding through social outings

WhatsApp works better than formal meetings	Confirmed Round 1; second quotation on the programme's potential value added to the same category in Round 2 as thematically continuous	Site B	D2 · WhatsApp as functional primary communication channel

D3.

Site A challenges aggregated from coded transcript summaries; Site B challenges (this domain and D1) share a workload/time thread flagged for cross-checking against D1 in Round 3 to avoid double-counting.

Working code / category (Round 1, independent coding)	Reconciliation note (Round 2 / Round 3)	Site(s)	Provisional mapping (subject to finalisation)
time constraints and competing workload	Aggregated pattern, no standalone excerpt	Site A	D3 · Time constraints and workload
students engage inconsistently	Aggregated pattern, no standalone excerpt	Site A	D3 · Variable student engagement
unsure how to handle sensitive student issues	Aggregated pattern, no standalone excerpt	Site A	D3 · Managing sensitive or complex student issues
no standard approach across faculty	Aggregated pattern, no standalone excerpt	Site A	D3 · Lack of standardisation
'another task squeezed in' / extra burden	Confirmed Round 1; cross-checked against D1 unclear-scope category in Round 3 and kept distinct (workload burden vs role clarity)	Site B	D3 · Workload / time burden
students disengaged, 'begging' students to attend	Confirmed Round 1; monosyllabic-response pattern noted at two further transcripts without separate excerpts	Site B	D3 · Student disengagement (begging metaphor; passive responses)
clashes with co-curricular commitments	Attributed pattern, no standalone excerpt	Site B	D3 · Co-curricular scheduling conflicts
feels artificial / compulsory, 'not supposed to be close friends'	Confirmed Round 1	Site B	D3 · Role ambiguity and programme artificiality
mentors personally funding refreshments, no institutional budget	Confirmed Round 1; corroborated at a second transcript in Round 2	Site B	D3 · Financial burden on faculty

D4.

Site A recommendations aggregated from coded transcript summaries and not yet matched to standalone excerpts; Site B recommendations largely single-source pending corroboration.

Working code / category (Round 1, independent coding)	Reconciliation note (Round 2 / Round 3)	Site(s)	Provisional mapping (subject to finalisation)
wants a standard operating procedure, logbook, defined schedule, periodic review	Aggregated pattern, no standalone excerpt	Site A	D4 · Structured monitoring framework
wants formal training including mental-health and case-simulation content	Aggregated pattern, no standalone excerpt	Site A	D4 · Formal training
wants certification, appraisal credit, incentives	Aggregated pattern, no standalone excerpt	Site A	D4 · Recognition and incentivisation
wants a digital / group communication platform	Aggregated pattern, no standalone excerpt	Site A	D4 · Digital and group communication platforms
wants better design and genuine mentor-mentee matching	Single-transcript origin; flagged for corroboration in Round 3, not found elsewhere, retained as a distinct recommendation rather than merged	Site B	D4 · Better programme design and genuine mentor-mentee matching
needs institutional backing (time, structure, resources)	Confirmed Round 1	Site B	D4 · Institutional administrative backing

D5.

Site A reflections aggregated from coded transcript summaries; Site B reflections explicitly conditional on programme quality, flagged in Round 2 as a distinct framing worth preserving rather than folding into a generic 'benefits' code.

**Working code / category (Round 1, independent coding)**	**Reconciliation note (Round 2 / Round 3)**	**Site(s)**	**Provisional mapping (subject to finalisation)**
finds mentoring professionally fulfilling	Aggregated pattern, no standalone excerpt	Site A	D5 · Professional fulfilment
improved own communication, empathy, patience	Aggregated pattern, no standalone excerpt	Site A	D5 · Improved communication skills, empathy, and patience
better understanding of what students face	Aggregated pattern, no standalone excerpt	Site A	D5 · Better understanding of the student environment
developed a sense of leadership identity	Aggregated pattern, no standalone excerpt	Site A	D5 · Development of leadership identity
benefits are real but conditional on the process being genuine and supported	Confirmed Round 1; wording of 'conditional' framing agreed in Round 2 after initial label 'mixed benefit' was felt to understate the participant's own qualification	Site B	D5 · Conditional benefits (only when genuine and supported)
confidence and communication gains when engagement is genuine	Confirmed Round 1, no standalone excerpt reported	Site B	D5 · Confidence and communication gains when genuinely engaged

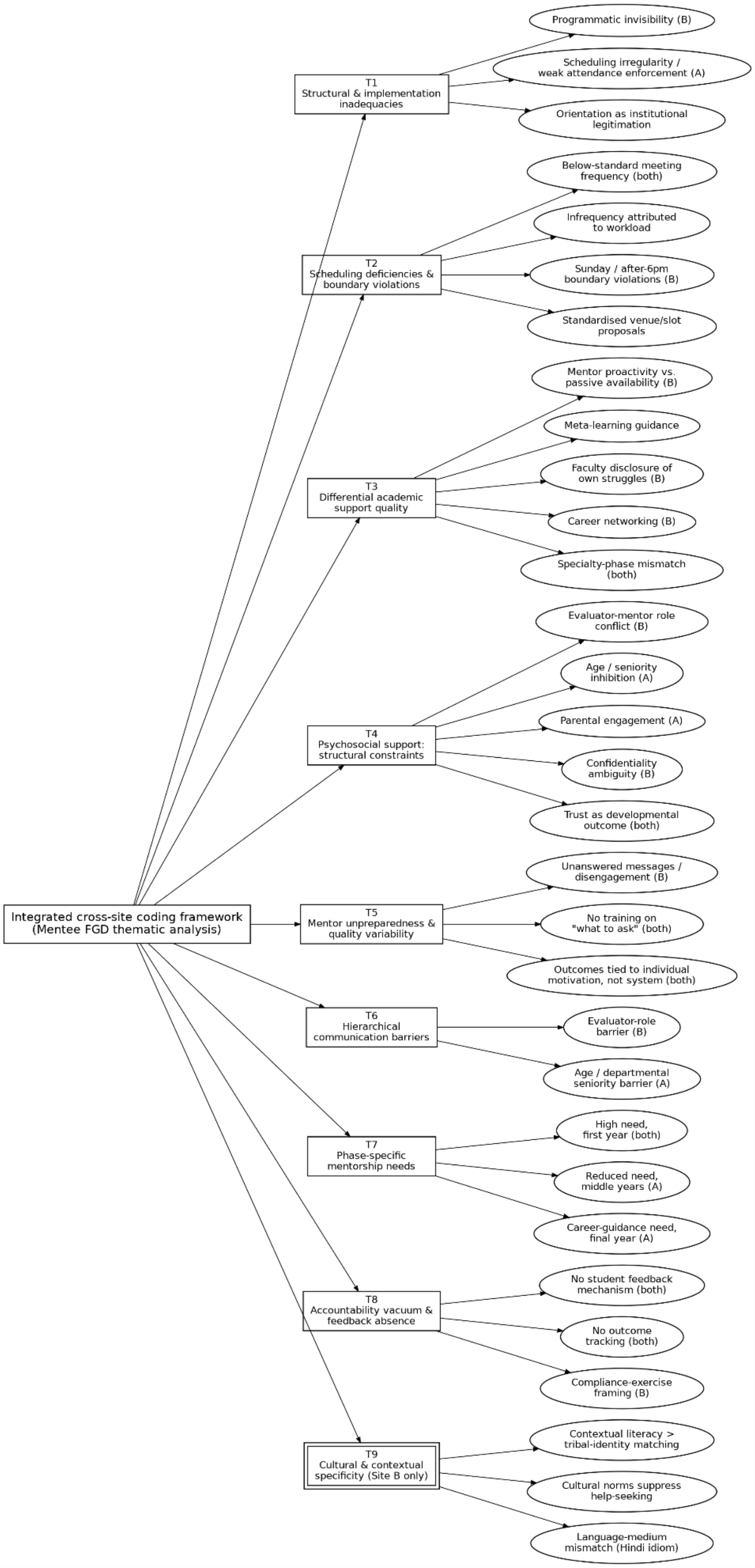
Integrated cross-site coding framework
(Mentee FGD thematic analysis)
T1
Structural & implementation inadequacies
Programmatic invisibility (B)
Scheduling irregularity / weak attendance enforcement (A)
Orientation as institutional legitimation
T2
Scheduling deficiencies & boundary violations
Below-standard meeting frequency (both)
Infrequency attributed to workload
Sunday / after-6pm boundary violations (B)
Standardised venue/slot proposals
T3
Differential academic support quality
Mentor proactivity vs. passive availability (B)
Meta-learning guidance
Faculty disclosure of own struggles (B)
Career networking (B)
Specialty-phase mismatch (both)
T4
Psychosocial support: structural constraints
Evaluator-mentor role conflict (B)
Age / seniority inhibition (A)
Parental engagement (A)
Confidentiality ambiguity (B)
Trust as developmental outcome (both)
T5
Mentor unpreparedness & quality variability
Unanswered messages / disengagement (B)
No training on "what to ask" (both)
Outcomes tied to individual motivation, not system (both)
T6
Hierarchical communication barriers
Evaluator-role barrier (B)
Age / departmental seniority barrier (A)
T7
Phase-specific mentorship needs
High need, first year (both)
Reduced need, middle years (A)
Career-guidance need, final year (A)
T8
Accountability vacuum & feedback absence
No student feedback mechanism (both)
No outcome tracking (both)
Compliance-exercise framing (B)
T9
Cultural & contextual specificity (Site B only)
Contextual literacy > tribal-identity matching
Cultural norms suppress help-seeking
Language-medium mismatch (Hindi idiom)

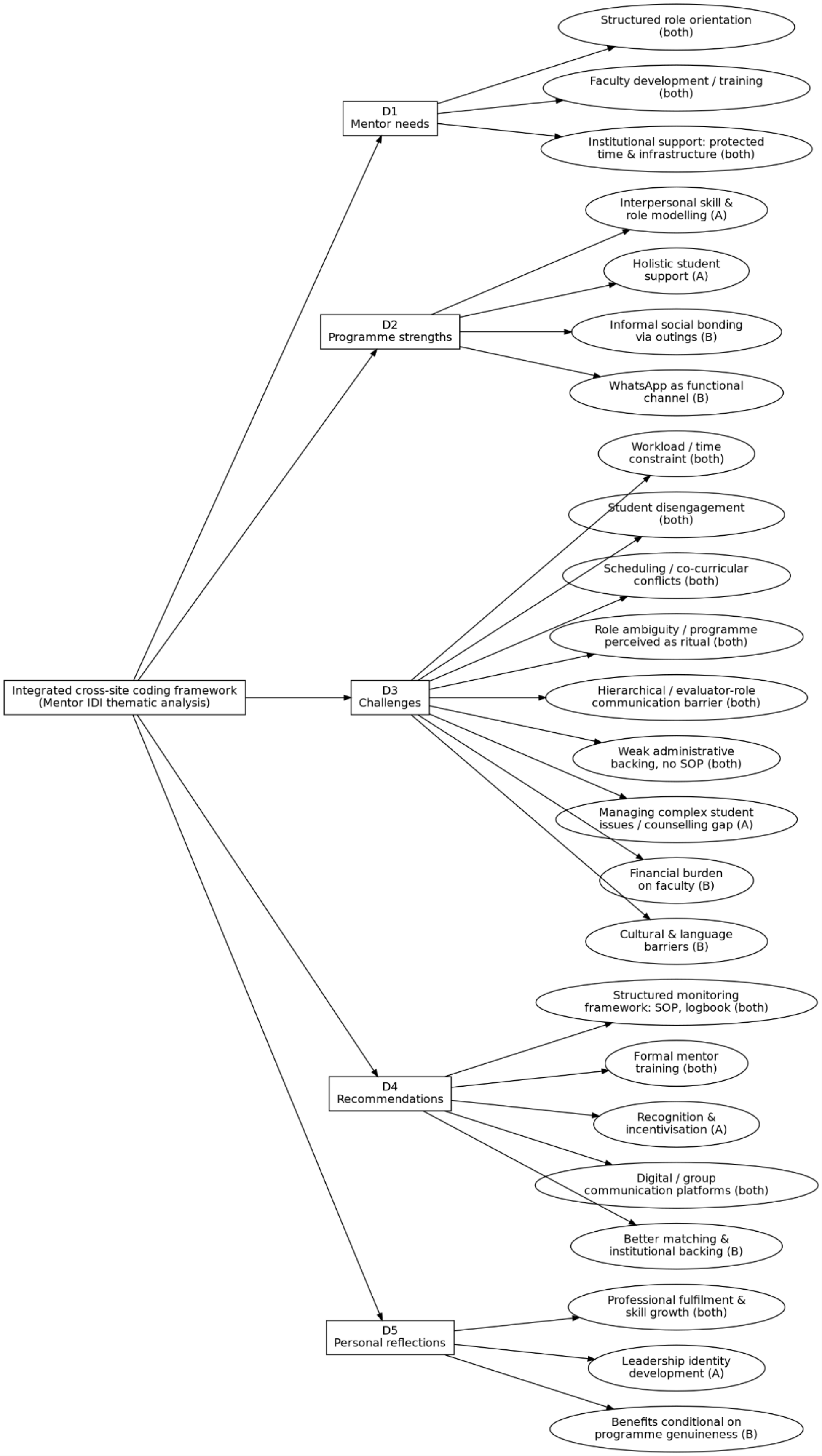
Integrated cross-site coding framework (Mentor IDI thematic analysis)
D1 Mentor needs
Structured role orientation (both)
Faculty development / training (both)
Institutional support: protected time & infrastructure (both)
D2 Programme strengths
Interpersonal skill & role modelling (A)
Holistic student support (A)
Informal social bonding via outings (B)
WhatsApp as functional channel (B)
D3 Challenges
Workload / time constraint (both)
Student disengagement (both)
Scheduling / co-curricular conflicts (both)
Role ambiguity / programme perceived as ritual (both)
Hierarchical / evaluator-role communication barrier (both)
Weak administrative backing, no SOP (both)
Managing complex student issues / counselling gap (A)
Financial burden on faculty (B)
Cultural & language barriers (B)
D4 Recommendations
Structured monitoring framework: SOP, logbook (both)
Formal mentor training (both)
Recognition & incentivisation (A)
Digital / group communication platforms (both)
Better matching & institutional backing (B)
D5 Personal reflections
Professional fulfilment & skill growth (both)
Leadership identity development (A)
Benefits conditional on programme genuineness (B)
